# A 10-Year Retrospective Study: Is Maternal Diabetes a Risk Factor for Associated Anomalies in Males With Cryptorchidism?

**DOI:** 10.7759/cureus.69892

**Published:** 2024-09-22

**Authors:** Mohamad Abdullah, Raed Al-Taher, Fadi Alhalasa, Firas K Jbareen, Edward S Nehme, Ahmad Kordi, Abdullah H AlHanbali, Nardin G Fadila, Mohammad Rasoul A Alqudah, Ahmad Aleliwi

**Affiliations:** 1 Pediatric Surgery, Royal London Hospital, Barts Health NHS Trust, London, GBR; 2 Pediatric Surgery, General Surgery Department, Jordan University Hospital, Amman, JOR; 3 Special Surgery, School of Medicine, University of Jordan, Amman, JOR; 4 Internal Medicine, School of Medicine, University of Jordan, Amman, JOR; 5 General Practice, School of Medicine, University of Jordan, Amman, JOR; 6 Anesthesia, Hamad Medical Corporation, Doha, QAT; 7 Pediatric Surgery, University Hospitals Sussex NHS Foundation Trust, Brighton, GBR

**Keywords:** cryptorchidism, gdm- gestational diabetes mellitus, genital anomaly, maternal diabetes mellitus, undescended testis

## Abstract

Background and objective

Cryptorchidism, or undescended testes (UDT), refers to the failure of one or both testicles to descend to their natural position in the scrotum of newborn males. The association of gestational diabetes with cryptorchidism has not been studied sufficiently. Hence, this study aimed to analyze the association between maternal diabetes and cryptorchidism and other associated anomalies among Jordanian male children.

Methods

We conducted a retrospective study involving women and their cryptorchid offspring over a 10-year period, between January 2010 and December 2019. The data were collected using a retrospective review of the hospital records and questionnaires administered via phone calls. We included all patients with cryptorchidism and excluded children with a diagnosis of disorders of sexual development.

Results

A total of 368 children with cryptorchidism were included, with 29 mothers suffering from gestational or type II diabetes at the time of pregnancy. About 25% of patients suffered from associated anomalies, the most common being genital anomalies, while hypospadias was the most common associated anomaly. We observed no differences in terms of the age of diagnosis or treatment of cryptorchidism between children born to diabetic and those born to nondiabetic mothers.

Conclusions

Diabetic mothers have a higher chance of having offspring with anomalies that might co-occur with cryptorchidism, particularly urogenital anomalies. Further research is needed to explore the link between maternal diabetes and these abnormalities. Screening for gestational diabetes is critical, and more extensive studies could provide clearer recommendations for screening protocols and early intervention.

## Introduction

Cryptorchidism, also known as undescended testes (UDT), is a condition where one or both testicles fail to descend to their natural position in the scrotum of newborn males. Isolated cryptorchidism is one of the most common congenital abnormalities of the male genital system, typically identified at birth. It affects 1-4% of full-term infants and is significantly more common in premature male infants, with an incidence rate of 15-30% [1-5]. However, this rate drops from 1.2% to 1.82% by the age of one year [1-5].

The incidence depends on age and location, with right-sided unilateral cryptorchidism being the most common presentation. Approximately 70% of UDT will descend spontaneously, usually by three months of age [1,4,6]. The exact cause of cryptorchidism is still largely unknown, as it is often unclear whether the condition is due to intrinsic testicular defects or an impairment in the descent process itself [3,4]. Also, the prevalence of congenital cryptorchidism and hypospadias varies significantly across regions. The Ministry of Health of Jordan publishes no data regarding the rate of UDT in Jordan. However, the rate of UDT in Jordan has been documented by multiple studies, ranging from 0.5% to 2.1% [6-8]. In Denmark, the current prevalence of cryptorchidism at birth is 9%, notably higher than the 3-5% reported in older boys in other countries [9].

The literature has reported extensively on the associations between pregnancies complicated with diabetes and specific congenital malformations [10-12]. However, only a few epidemiologic studies have evaluated the association between gestational diabetes and the risk of cryptorchidism [4]. This study aims to investigate the association between maternal diabetes and associated anomalies in Jordanian male babies born with cryptorchidism. We believe our findings will help assess the association, prevalence, and risk factors, thereby aiding in raising awareness among healthcare providers and the general population, ultimately reducing the complications associated with cryptorchidism.

## Materials and methods

A retrospective study was conducted involving women and their male offspring diagnosed with undescended testis in a tertiary teaching hospital in Jordan between January 2010 and December 2019. Data regarding maternal diabetes status were obtained from hospital records for mothers who had follow-ups at the hospital or through a phone questionnaire. Data on males with UDT were collected from hospital records, including gestational age, birth weight, age at diagnosis, other anomalies with undescended testis, age of surgery, and laterality of cryptorchidism. Telephone-based questionnaires were utilized to address any missing data. This study received approval from the School of Medicine's ethical committee.

Children diagnosed with UDT between the ages of one day and 12 years were included. Data regarding family history, site of testicular descent, prematurity, mode of delivery, maternal age at the time of conception, and presence of other associated anomalies were also collected. Patients diagnosed with disorders of sexual development were excluded from this study.

Data were analyzed using SPSS Statistics version 25.0 (IBM Corp., Armonk, NY). Descriptive statistics, including means and standard deviations (SD), were calculated for continuous variables such as gestational age. Frequencies were computed to describe other nominal variables, such as the side of UDT. Chi-square and Fisher's exact tests were employed to determine the relationships between variables. All underlying assumptions were met unless otherwise indicated. We assigned a p-value of 0.05 as the significance cut-off point. Due to a violation of the normality assumption, we used the Mann-Whitney U test to assess differences in gestational age at birth, age at diagnosis of cryptorchidism, and age at orchidopexy surgery between infants of diabetic and nondiabetic mothers.

## Results

Patient characteristics

A total of 368 children, aged one day to 12 years, with gestational age at birth of 26 to 40 weeks (mean: 37.9 weeks), were included in this study; the mean age at diagnosis was 28.0 ± 33.5 months, and the mean age at orchidopexy surgery was 43.0 ± 33.5 months. Twenty-nine children with UDT (7.9%) were born to a diabetic mother. The percentage of patients with bilateral cryptorchidism was 37.2%, and 23.1% had a positive family history of cryptorchidism. There was no difference in gestational age at birth (p=0.241), age at diagnosis of cryptorchidism (p=0.859), and age at orchidopexy surgery (p=0.459) between diabetic and nondiabetic mothers. Detailed characteristics of patients born with undescended testis are presented in Table 1. 

**Table 1:** Characteristics of patients born with undescended testis for both diabetic and nondiabetic mothers

Characteristics	Variables	Number (%)
Birth weight, kg	Very low: <1.5	15.0 (4.1)
	Low birth weight: 1.5-2.5	42.0 (11.4)
	Normal: 2.5-4.5	310.0 (84.2)
	Overweight: >4.5	1.0 (0.3)
Gestational age	Term	316 (85.9)
	Preterm	35 (9.5)
	Post-term	17 (4.6)
Age range at diagnosis	Neonatal period: 0-4 weeks	61 (16.6)
	Infancy: 1-12 months	117 (31.8)
	Early childhood: 1-6 years	162 (44.0)
	Late childhood: 7-12 years	25 (6.8)
	Adolescence: 13-20 years	3 (0.8)
Laterality of cryptorchidism	Bilateral	137 (37.2)
	Left	119 (32.3)
	Right	112 (30.4)
Age range at orchiopexy surgery, years	≤1	90 (24.5)
	1-6	193 (52.4)
	>6	85 (23.1)
Presence of family history	Yes	85 (23.1)
	No	283 (76.9)
Specified family history of undescended testes	Brother(s)	40 (10.9)
	Father	4 (1.1)
	Grandfather	1 (0.3)
	Maternal uncle	8 (2.2)
	Paternal uncle	7 (1.9)
	Maternal or paternal cousin(s)	25 (6.8)

Relationship between maternal diabetes and type of anomalies associated with UDT

Upon further evaluation of associated anomalies in children with UDT, it was found that patients with associated anomalies were more likely to have a diabetic mother (24.1%) compared to patients without other anomalies (2.8%). Additionally, children with two or more anomalies had a higher likelihood of having a diabetic mother (19.7%) than those with just one other anomaly (40%): χ2=36.72, p<0.001 (Table 2).

**Table 2:** Detailed differences in continuous variables between patients with undescended testis born to diabetic and nondiabetic mothers

Variable	Maternal diabetes status	N (%)	Median	U	P-value
Gestational age at birth, weeks	Diabetic	29 (7)	38.0	4316.5	0.241
	Not diabetic	339 (93)	38.0		
Age at undescended testis diagnosis, months	Diabetic	29 (7)	24.0	4817.0	0.859
	Not diabetic	339 (93)	16.0		
Age at orchidopexy surgery, months	Diabetic	29 (7)	36.0	4507.5	0.459
	Not diabetic	339 (93)	30.0		

Of note, 48% of mothers of cryptorchid children were aged 25-35 years at the time of pregnancy, and 42% were 35-50 years. Only two types of maternal diabetes were found: type 2 diabetes and gestational diabetes. The number of mothers who had diabetes was 29 (19 gestational diabetes and 10 type II diabetes mellitus). Of mothers with diabetes, 6% were well-controlled, and 2% were poorly controlled in our study population.

Mothers who smoked during pregnancy had a higher chance of having a child with cryptorchidism; to illustrate, among the mothers who gave birth to boys with cryptorchidism, 26.1% were smokers, of which 4.1% were active smokers and 22.0% were passive smokers. Detailed characteristics of mothers included in this study are shown in Table 3.

**Table 3:** Characteristics of diabetic mothers with babies born with undescnded testis HbA1c: hemoglobin A1c

Mothers' characteristics	Variables	Frequency	%
Mother's age group at the time of pregnancy, years	15-25	39	10.6%
	25-35	176	47.8%
	35-50	153	41.6%
Mother's educational level	Below high school	46	12.5%
	High school graduate	83	22.5%
	College	186	50.5%
	Missing	53	14.4%
Smoking status	Yes	96	26.1%
	No	272	73.9%
Smoking activity	Active smoking	15	4.1%
	Passive smoking	81	22.0%
	Not smoker	272	73.9%
Parity	≤5	325	88.3%
	≥6	43	11.7%
	1	41	11.1%
	2	52	14.1%
	3	90	24.5%
	4	94	25.5%
	5	48	13.0%
In-vitro fertilization	Yes	10	2.7%
	No	358	97.3%
Maternal diabetes	Yes	29	7.9%
	No	339	92.1%
Type of diabetes	Gestational	19	5.2%
	Type 2	10	2.7%
Diabetes control	Poorly controlled	7	1.9%
	Well controlled	22	6.0%
HbA1c during pregnancy	Below 5.7	1	0.3%
	5.7-6.4	13	3.5%
	6.5 and above	7	1.9%
	Not available	8	2.2%

Characteristics and type of anomalies associated with UDT

UDT was analyzed concerning other anomalies and their association with it. Anomalies studied included anomalies of the external genitalia, urinary tract and kidney, cardiac, respiratory, musculoskeletal, and central nervous system. To note, of children diagnosed with UDT, 32.6% had other anomalies, with those related to the genital system being the most common (9.0%); hypospadias was the most common associated anomaly (7.1%), followed by those related to the musculoskeletal system (5.7%) (Table 4).

**Table 4:** Other anomalies associated with cryptorchidism in our patients UDT: undescended testes

Associated anomalies with UDT	Variables	Frequency	%
Presence of other associated anomalies	Yes	87	23.6%
	No	281	76.4%
Number of associated anomalies in each patient	One associated anomaly	61	16.6%
	Two associated anomalies	11	3.0%
	Three or more associated anomalies	9	2.5%
Cardiac and respiratory system anomalies	Total	9	2.4%
	Ventricular septal defect	5	1.4%
Central nervous system anomalies	Total	12	3.3%
Urinary system anomalies	Total	12	3.3%
	Kidney anomalies	5	1.4%
Genital system anomalies	Total	33	9.0%
	Hypospadias	26	7.1%
	Disorder of sexual differentiation	5	1.4%
Gastrointestinal system anomalies	Total	6	1.6%
Musculoskeletal and skin system anomalies	Total	21	5.7%
	Hernia	15	4.1%
	Umbilical hernia	4	1.1%
	Diaphragmatic hernia	5	1.4%
	Inguinal hernia	7	1.9%
Laterality of inguinal hernia	Bilateral	2	0.5%
	Left	2	0.5%
	Right	3	0.8%
Chromosomal abnormality	Total	5	1.4%
	Trisomy	3	0.8%

Furthermore, all children with urinary tract and kidney anomalies were born to diabetic mothers. Two-thirds of the children with genital anomalies were born to diabetic mothers, compared to only 5.7% of patients without genital anomalies. Conversely, none of the children with cardiac, respiratory, or central nervous system anomalies were born to diabetic mothers. The chi-square test of independence showed a significant association between maternal diabetes and the presence of UDT with its associated anomalies, x^2^(1)=41.48, p<0.001.

Moreover, Fisher's exact test indicated significant associations between maternal diabetes and various anomalies. Specifically, there were significant associations with urinary system anomalies (p<0.001), kidney anomalies (p<0.001), genital system anomalies (p<0.001), hypospadias (p<0.001), musculoskeletal system anomalies (p=0.018), hernia (p=0.023), and umbilical hernia (p=0.002). Notably, hypospadias and umbilical hernia were present in 34.6% and 75.0% of infants born to diabetic mothers, respectively (Table 5).

**Table 5:** Analysis of various factors associated with maternal diabetes mellitus and associated anomalies with UDT (chi-square test and Fisher's exact test) ^*^Cramér’s V. ^**^Fisher's exact test. ^†^Statistically significant UDT: undescended testes

Variable	Level	Children with a diabetic mother, n (%)	Pearson's chi-square	Exact significance (two-sided)	Phi or Cramér’s V*
Mode of delivery			0.195	0.696	0.023
	Vaginal	19 (8.4%)			
	Cesarean section	10 (7.1%)			
Birth weight, kg			0.069	1.00	-0.013
	≥2.5	25 (8.0%)			
	Low (<2.5)	4 (7.0%)			
Birth age			1.34	0.339	0.060
	Term	28 (8.4%)			
	Preterm	1 (2.9%)			
Age range at diagnosis, years			1.22	0.572	0.058*
	<1	13 (7.3%)			
	1-6	15 (9.3%)			
	>7	1 (3.6%)			
Laterality of cryptorchidism			0.792	0.693	0.046*
	Bilateral	13 (9.5%)			
	Right side	8 (7.1%)			
	Left side	8 (6.7%)			
Age range at orchiopexy surgery, years			2.298	0.334	-0.026*
	>1	6 (6.7%)			
	1-6	13 (6.7%)			
	>7	10 (11.8%)			
Presence of family history			0.103	0.823	-0.017
	Positive	6 (7.1%)			
	Negative	23 (8.1%)			
Mother's age group at the time of pregnancy, years			2.39	0.168	-0.081
	<35	13 (6.0%)			
	>35	16 (10.5%)			
Smoking status			2.46	0.128	-0.082
	Smoker	4 (4.2%)			
	Non-smoker	25 (9.2%)			
Smoking activity			2.89	0.236	0.089
	Active	0 (0.0%)			
	Passive	4 (4.9%)			
	Non-smoker	25 (9.2%)			
Parity			0.055	1.00	0.012
	≤5	26 (8.0%)			
	>5	3 (7.0%)			
Presence of other associated anomalies			41.48	<0.001^†^	0.336
	Yes	21 (24.1%)			
	No	8 (2.8%)			
Number of associated anomalies in each patient			36.72	<0.001^†**^	0.365*
	None	8 (2.8%)			
	One other anomaly	13 (19.7%)			
	Two or more other anomalies	8 (40.0%)			
Cardiac and respiratory system anomalies			-	1.00^**^	-0.046
	Present	0 (0.0%)			
	Absent	29 (8.1%)			
Central nervous system anomalies			-	0.610^**^	-0.054
	Present	0 (0.0%)			
	Absent	29 (8.1%)			
Urinary system anomalies			-	<0.001^†**^	0.628
	Present	12 (100.0%)			
	Absent	17 (4.8%)			
Kidney anomalies			-	<0.001^†**^	0.401
	Present	5 (100.0%)			
	Absent	24 (6.6%)			
Genital system anomalies			-	<0.001^†**^	0.261
	Present	10 (30.3%)			
	Absent	19 (5.7%)			
Disorders of sexual development			-	0.338^**^	0.053
	Present	1 (20.0%)			
	Absent	28 (7.7%)			
Hypospadias			-	<0.001^†**^	0.274
	Present	9 (34.6%)			
	Absent	20 (5.8%)			
Gastrointestinal system anomalies			-	0.074^**^	0.122
	Present	2 (6.9%)			
	Absent	27 (7.5%)			
Musculoskeletal system anomalies			-	0.018^†**^	0.145
	Present	5 (23.8%)			
	Absent	24 (6.9%)			
Hernia			-	0.023^†**^	0.144
	Present	4 (26.7%)			
	Absent	25 (7.1%)			
Umbilical hernia			-	0.002^†**^	0.261
	Present	3 (75.0%)			
	Absent	26 (7.1%)			
Diaphragmatic hernia			-	0.052^**^	0.140
	Present	2 (40.0%)			
	Absent	27 (7.4%)			
Inguinal hernia			-	1.000^**^	-0.041
	Present	0 (0.0%)			
	Absent	29 (8.0%)			
Chromosomal abnormality			-	1.000^**^	-0.034
	Present	0 (0.0%)			
	Absent	29 (8.0%)			

A post-hoc test using Phi/Cramer's V revealed that the strongest association between maternal diabetes and anomalies in cryptorchid children manifested in urinary system anomalies (Phi=0.628), followed by kidney anomalies (Phi=0.401). The number of associated anomalies per patient (V=0.365) and other associated anomalies (Phi 0.336) also showed significant associations.

## Discussion

The frequency of diabetes in females of childbearing age has been increasing for the last two decades, with approximately 1% of all pregnancies now reported to have it. Pre-gestational diabetes mellitus [type 1 or type 2 diabetes mellitus (T1DM or T2DM)] may result in several adverse effects on the offspring. Diabetes during pregnancy, whether gestational or pre-gestational, could impact the child, neonate, and even the fetus [13-14]. Pre-gestational diabetes is associated with major birth defects in 5-10% of pregnancies and spontaneous abortion in 15-20% of pregnancies [13,15,16]. The cause of this complication is still unknown, but hormonal changes and placental dysfunction due to poor glycemic control may be an inciting factor. During genital development in early pregnancy, insufficient androgen may result in multiple genital anomalies [13,14,17] In 2001, Skakkebæk et al. proposed a theory that certain male reproductive disorders are interconnected and that a form of abnormal testicular development during fetal life causes this disorder. This theory was named testicular dysgenesis syndrome (TDS) [18]. Mo et al. studied mice induced with diabetes serum levels and found that gene development is affected, resulting in disrupted testicular development [19].

Brouwers et al. found no association between maternal diabetes and cryptorchidism [20]. However, many authors have confirmed that maternal diabetes is a risk factor for UDT in the offspring [21]. Virtanen et al. found that in the group of cryptorchid boys, 10.4% of the mothers had diabetes, and they all had diet-treated gestational diabetes. In comparison, 5.6% had abnormal results in the OGTT without a diagnosis of diet-treated gestational diabetes [22]. In our study, the percentage of diabetic mothers who gave birth to cryptorchid sons was 7.9%, of which 5.2% had gestational diabetes and 2.7% had type 2 diabetes. This is thought to be due to decreased levels of serum sex hormone-binding globulin (SHBG) and increased levels of insulin in umbilical cord blood in pregnant women with diabetes, which interfere with testicular descent [14,17,23].

It has been reported that boys whose mothers had type 1 diabetes were more prone to cryptorchidism and hypospadias [hazard ratio (HR) for hypospadias=2.33, 95% confidence interval (CI): 1.48, 3.66; and HR for cryptorchidism=1.92, 95% CI: 1.39, 2.65]. There was a slightly higher risk for boys born to mothers with gestational diabetes. The highest incidence was seen in boys whose mothers had diabetic complications before pregnancy, indicating that poorly managed diabetes poses an extra risk [14]. In this study, we included 368 children with unilateral or bilateral cryptorchidism, of which 29 mothers had gestational or type II diabetes at the time of pregnancy. About 25% of patients had other anomalies; the genital system was most commonly affected, with hypospadias being the most commonly associated anomaly. We observed no difference in age of diagnosis or treatment of cryptorchidism between children born to diabetic or nondiabetic mothers. Children of diabetic mothers had a higher chance of having two or more anomalies in the urogenital and musculoskeletal system, which include kidney anomalies, hypospadias, hernias, and umbilical hernias.

We also found that cryptorchidism was bilateral in 37.2% of cases and unilateral in 62.8%. These findings are consistent with a study indicating that unilateral cryptorchidism is more than twice as common as bilateral, with the right side being affected more often than the left (70% compared to 30%) [1]. However, our study results differ, showing that 32.3% of cases were on the left and 30.4% on the right. In our study, we found that of the mothers who gave birth to boys with cryptorchidism, 26.1% were smokers, of which 4.1% were active smokers and 22.0% were passive smokers. In contrast, another study showed that mothers who smoked during pregnancy had a 1.17 times higher chance of having a child with cryptorchidism than mothers who did not smoke during pregnancy. This is thought to be due to an association between smoking and decreased levels of HCG, which is needed for testicular descent. Another theory states that smoking reduces paternal semen quality, which leads to cryptorchidism in the newborn [20,21,22,24].

There is an association between higher maternal age at the time of pregnancy and the delivery of babies with UDT. This is described by Brouwers MM et al., who showed that 14% of the cases of cryptorchidism were associated with maternal age >35 years [23]. Another study found that 8.8% of newborns with congenital cryptorchidism were related to maternal age >39 years at the time of delivery [23]. On the other hand, in our study, we noticed that 42% of mothers with children with cryptorchidism were >35 years of age at the time of pregnancy. Genetic predisposition factors could alternate the risk of cryptorchidism in newborns among mothers from different races within the same age group.

Trabert et al. have stated that Insulin usage among women diagnosed with gestational diabetes had an associated risk of cryptorchidism of 12%, which is very low compared to non-insulin usage among women with gestational diabetes (88%) [4]. This has been confirmed by other reports [3,13,22]. Of mothers with diabetes, 6% were well-controlled, and 2% were poorly controlled in our study population. We might need a larger sample size to confirm that controlling gestational diabetes with insulin could significantly decrease the risk of cryptorchidism. Thus, appropriate patient counseling should be conducted [4]. It is worth mentioning that women without previous live births are at an increased risk for nearly all male genital malformation phenotypes in offspring (hypospadias, second- or third-degree hypospadias, small penis, and cryptorchidism) [2,3,21,25]. Our results showed that primiparous mothers had an 11% associated anomaly with UDT. However, this rate increases as the mother gives birth to more children (24.5% in three, 25.5% in four, and 13% in five previous pregnancies).

Cryptorchidism was observed in around 1.5% to 4% of fathers and 6% of brothers of individuals with the condition. Studies suggest that the heritability in first-degree male relatives is estimated to be approximately 0.5% to 1%. Furthermore, 7% of siblings of boys with undescended testes also have cryptorchidism, highlighting its potential genetic predisposition within families [22,26,27]. Regarding family history in our study, we found that 23.1% of the patients had a positive family history of cryptorchidism. Of patients with a positive family history, we noticed that brothers (10.9%) were the most common link, which may indicate a genetic association. Maternal or paternal cousins came next (6.8%), followed by maternal uncles (2.2%), paternal uncles (1.9%), fathers (1.1%), and finally, grandfathers (0.3%).

Cryptorchidism has been distinctly associated with low birth weight and gestational age [24,28]. A study by Jensen et al. involving 934,538 Danish boys reported an increase in the risk of cryptorchidism in boys who were born preterm or born with low birth weight [21,28]. In our study, the mean gestational age at birth was 37.9 weeks; most children were born at term (n=316), birth weight was average (n=310), and its association with cryptorchidism was 85.9% and 84.2%, respectively. Moreover, we noticed that children born preterm (n=35) or those with low birth weight (n=57) had a 9.5% and 15% association with cryptorchidism, respectively. These results indicate that newborn characteristics could increase the risk of cryptorchidism occurrence. At the same time, the prenatal circumstances that can lead to inadequate fetal growth also increase the risk of cryptorchidism, which may have a causative role. Our results showed that only 10 children (2.7%) were the product of in-vitro fertilization (IVF) conception, which does not show a significant correlation. This finding is endorsed by another study that showed no adverse effect of IVF on cryptorchidism [29].

Our study has certain limitations. A larger sample size could provide deeper insights into the association (or lack of it) between cryptorchidism and diabetic pregnant women. We did not depend entirely on the hospital's records, as we had to use the telephone to talk with the mothers, which might have introduced recall bias. Our study did not include a control group of infants without cryptorchidism. This limits our ability to draw firm conclusions about potential links between maternal diabetes and cryptorchidism and any other anomalies associated with cryptorchidism. It is important to note that even with a control group, this study would only allow us to observe associations, as the design does not involve a randomized controlled trial and, therefore, cannot establish causality.

## Conclusions

Diabetic mothers have a higher chance of having offspring with any other anomalies that might co-occur with cryptorchidism, especially in the urogenital system. Our study underscores the need for further investigations into the possible link between maternal diabetes and urogenital abnormalities. It also stresses the importance of recognizing the risks associated with maternal diabetes and implementing screening for gestational diabetes. Further studies with extensive data could provide clearer recommendations for screening protocols for anomalies in infants of diabetic mothers and contribute to early intervention when necessary.
